# k-Tournament Grasshopper Extreme Learner for FMG-Based Gesture Recognition

**DOI:** 10.3390/s23031096

**Published:** 2023-01-18

**Authors:** Rim Barioul, Olfa Kanoun

**Affiliations:** Chair of Measurement and Sensor Technology, Technische Universitat Chemnitz, 09126 Chemnitz, Germany

**Keywords:** extreme learning machine, force myography, grasshopper optimization algorithm, k-tournament selection

## Abstract

The recognition of hand signs is essential for several applications. Due to the variation of possible signals and the complexity of sensor-based systems for hand gesture recognition, a new artificial neural network algorithm providing high accuracy with a reduced architecture and automatic feature selection is needed. In this paper, a novel classification method based on an extreme learning machine (ELM), supported by an improved grasshopper optimization algorithm (GOA) as a core for a weight-pruning process, is proposed. The k-tournament grasshopper optimization algorithm was implemented to select and prune the ELM weights resulting in the proposed k-tournament grasshopper extreme learner (KTGEL) classifier. Myographic methods, such as force myography (FMG), deliver interesting signals that can build the basis for hand sign recognition. FMG was investigated to limit the number of sensors at suitable positions and provide adequate signal processing algorithms for perspective implementation in wearable embedded systems. Based on the proposed KTGEL, the number of sensors and the effect of the number of subjects was investigated in the first stage. It was shown that by increasing the number of subjects participating in the data collection, eight was the minimal number of sensors needed to result in acceptable sign recognition performance. Moreover, implemented with 3000 hidden nodes, after the feature selection wrapper, the ELM had both a microaverage precision and a microaverage sensitivity of 97% for the recognition of a set of gestures, including a middle ambiguity level. The KTGEL reduced the hidden nodes to only 1000, reaching the same total sensitivity with a reduced total precision of only 1% without needing an additional feature selection method.

## 1. Introduction

Hand gestures are part of behavioral attributes that are authentic (emphasize or help to express a thought or feeling), distinguishable (present a known meaning that depends on culture, language, and use case), and have unique physiological patterns (physiological signals and phenomena resulted from various hand gestures present varying unique properties). Hand gesture recognition is essential in several applications, such as sign language, mobile security systems, smart homes, and other IoT-based applications. In addition, hand gesture recognition involves several challenges concerning the sensors and machine learning algorithms, including the system design, which needs to fit different persons, and the influence of the physiological state of the subject on the collected signal quality. Hand grasp recognition and hand sign recognition are the main subtopics of hand gesture recognition. The first is dedicated to the identification of the grasping nature, and the object-handling tasks while sign recognition is dedicated mainly to communication between persons or between persons and intelligent agents. Hand sign recognition is valuable, e.g., for communication over long distances, in noisy environments, and with people with disabilities. Identifying hand signs with camera-based systems is challenging in such environments and suffers from limited resolution, significant distances, and varying light conditions. Myographic measurement methods and sensors, which allow the direct collection of information on the muscle state during the gesture performance, can be of great importance in overcoming these limitations. Techniques such as surface electromyography, force myography, and surface electrical impedance myography show promising performance for gesture detection, even if only a few current investigations exist for sign language recognition based on myographic signals. Another challenge for hand sign classification is in the level of algorithms and features. The classification algorithm must get a suitable feature subset to be able to realize a high classification accuracy. In addition, the quality of the classification is variable, along with the number of features. Hence, the control of the feature number is essential since a limited feature number may cause data overlapping, which means that the classification becomes not sufficiently grounded. Too many features increase the dimension of the problem, and more complex classification algorithms will be needed. Thus, the goal of feature selection is to define the best subset of features by directly removing the irrelevant and redundant features from the data and improving the classification performance and stability. Moreover, reduced resource consumption is required to ensure the suitability of the classification algorithm with wearable hand gesture recognition systems. Most investigations adopt a feature selection based on metaheuristic optimization methods in binary format. The classification accuracy depends on many factors, including the gesture types and numbers, the measurement accuracy of the myographic signals, and the choice of the classifier itself. Furthermore, the classification method should be suitable for solving multiclass problems with minimal calculation. Such property is reported to be insured by an extreme learning machine (ELM). It is a single-layer feed-forward network (SLFN) with randomly generated input weights and biases and output layer weights calculated via linear algebra methods allowing fast training in only one iteration, even in multiclassification problems. However, ELM suffers from the incertitude caused by this random weight generation. Many optimization methods have been suggested in the literature to solve this problem, including controlling the randomization and pruning the hidden nodes. However, weight pruning is not sufficiently investigated for the ELM architecture’s optimization. This work proposes a new approach for ELM network optimization based on a coupled weight and feature selection that allows not only the elimination of irrelevant weights in the network but also an integrated feature selection and hidden node number reduction.

The paper is structured as follows: In Section 2, related works are described, which provide information on the state of the art of ELM pruning and FMG-based gesture recognition. In Section 3, the methodology of implementation of a k-tournament grasshopper extreme learner, the ELM weight selection concept, and the proposed KTGEL is detailed. Section 4 shows the study of the number of FMG sensors for an efficient hand sign recognition system and the influence of the number of subjects on the KTGEL performance. This section also provides the experimental investigation on the performance of the KTGEL compared with the state of the art and with a variation of the ambiguity level in the data set followed by the conclusion.

## 2. Related Work

In the first part of this section, we present an overview of applied methods for pruning an extreme learning machine to reduce its model architecture while keeping its good performance and exposing the gap in approaches exploited to fulfill this aim. In the second part, an overview of hand gesture recognition based on FMG sensors is presented, focusing on the number of sensors, the features, the number of subjects, and the American Sign Language recognition as an application.

### 2.1. Pruning of Extreme Learning Machine

An extreme learning machine (ELM) is a single-layer feed-forward network (SLFN) where the fundamental concept is that the weights and biases of the hidden layer are randomly generated. Moreover, the output layer weights are calculated using a least-squares solution defined by the outputs of the hidden layer and the target [1]. Thus, the weights that connect the hidden nodes to the outputs can be trained very fast in one iteration according to the pseudocode presented in Algorithm 1.
**Algorithm 1:** Pseudocode of an extreme learning machine [2].1. Given a training set $N=\left\{\left(x_{i},t_{i}\right)|x_{i}\in R^{n},t_{i}\in R,i=1\cdots N\right\}$, activation function G(w,b,x), and number of hidden nodes $\tilde{N}$;2. Assign random input weights $w_{i}$, and biases $b_{i}$, for $i=1\cdots \tilde{N}$;3. Calculate the hidden layer output matrix H;4. Calculate the output weight matrix
(1)$$\beta =H^{†}T$$ where $H^{†}$ is the Moore–Penrose generalized inverse of matrix H and $T={\left[t_{1}\cdots t_{N}\right]}^{T}$; The output weight matrix β;

Since its first introduction, the ELM has been a subject for optimization as it represents a promising possibility for embedded systems and online real-time classification tasks. However, it also presents some limitations, especially in its hidden node number and weights’ randomization method. An ELM also randomly generates the input weights and the bias of hidden nodes, which has the following consequences: first, a slow learning speed caused by the minor roles played by some hidden nodes with too small output weights on the network’s output; second, a slow error reduction during the training process is caused by these invalid hidden layer neurons, which increase the network complexity [3]. To solve this, most of the proposed algorithms focus on simplifying the computation process, finding the optimized depth of the SLFNs, or expanding the range of the generalized methods via multilayers or a complex domain. However, for random weight optimization, the proposed solutions tend to replace the completely randomly generated input weight and bias with fully controllable metrics, which turns the ELM into a controlled method and reduces the benefits of the weights’ randomness in the ELM results. The optimally pruned ELM (OP-ELM) was proposed by Miche et al. [4] based on the ELM algorithm in terms of kernel selection and using the methodology of pruning the neurons, leading to more efficient algorithms and improving the ELM problems experienced when using irrelevant or correlated data [4,5]. Compared to the ELM, the OP-ELM enhanced the robustness and accuracy of the network. However, it had a higher computational time, affecting the accuracy and training time [6,7]. Genetic algorithms for pruned ELM (GPA-ELM) were proposed by Alencar et al. [8] to prune the hidden layer neurons based on multiobjective GAs. It combined the advantages of ELMs and GAs to optimize the performance of the ELM classifiers and prune the maximum possible number of hidden neurons. In [9], the authors proposed the PSO-ELM for optimizing the input feature subset selection and the number of hidden nodes to enhance the classification performance of ELM in the application of power system disturbances classification. The experimental results showed that the proposed PSO-ELM was faster and more accurate than the original ELM algorithm. However, the PSO which was used to perform those optimizations was reported to be outperformed by other newly introduced swarm intelligence optimization methods, including the GOA [10,11,12]. In the literature, the main difference between the various pruned ELM versions is the different optimization methods implemented to modify the ELM architecture to realize the hidden nodes’ pruning. However, there is no specific idea proposed so far about weight selection without controlling the random initialization or connection pruning optimization, which is an integral part of extreme machine learning in data classification. Hence, in this work, optimizing the ELM by proposing a weight selection by an improved version of the GOA after the initial random initialization is presented as a methodology for connection pruning in ELMs.

### 2.2. Sensors for FMG

Since it is possible to perform FMG with either pressure or strain sensors, unlimited choices of sensors are available. However, in 2006, Amft et al. [13] compared the force-sensitive resistor (FSR) as a pressure sensor with a fabric stretch sensor (FSS) as a strain sensor and surface electromyography (sEMG) for monitoring muscles’ contraction for grasping, upper-hand activities, and object lifting. The feasibility of muscle activity detection by the strain and pressure sensors as alternatives to sEMG was confirmed in that study. Moreover, the experimental results showed that the pressure sensors were more suitable as a future alternative to sEMG for gesture recognition applications as they were able to monitor the contraction of more muscle groups than the strain sensor. Hence, the FSR pressure sensor for FMG measurement was chosen for this study. Moreover, as commercial sensors were more suitable for this work’s aims, a study of the FSR sensors market and publications was conducted. As the cost for various FSR sensors were almost similar, and the FSR sensor by Interlink Electronics and the FlexiForce™ by Tekscan Ink were the most popular commercial sensors, which were used in 55% of publications about FMG applications until 2019 [14], the sensor choice range was limited between both these sensors. Their characteristics extracted from their data sheets are shown in Table 1.

Vecchi et al. compared the previous sensors on several points, such as repeatability, time drift, or dynamic force measurement via an experimental process. The results showed that the FlexiForce sensors had better performance in terms of linearity, repeatability, time drift, and dynamic accuracy. However, Interlink’s FSR was more robust [15]. Another study that compared the same sensors with the LuSense PS3 (Standard 151) sensor was conducted in 2006 and concluded that the FlexiForce had not only the highest precision but also the highest noise with the slowest response time and the highest resistance dropping from the nominal value during subsequent tests [16]. Hence, each sensor has its pros and cons. The choice was based on the response time as a real-time and fast system was the goal in this study’s outlook. Thus, the Interlink’s FSR possessing the lowest response time in the data sheet (see Table 1) and in experiments [16] was chosen to perform the FMG data collection in this work. A typical Interlink Electronics’s FSR sensor consists of a top carbon-based ink layer and a bottom conductive substrate layer with a spacer adhesive located in the middle of the two layers [17]. Therefore, during FMG collection, as the hand exerts a force, the corresponding muscles on the arm produce a deformation on the skin’s surface. These deformations apply pressure to the surface of the top layer of the FSR, changing its resistance. These changes in resistance can be translated into corresponding changes in voltage by a voltage divider structure resulting in the FMG distinct patterns that could be used for hand gesture recognition with the best sensitivity, which is ensured by a reference resistance of 100 kΩ in the voltage divider [17,18].

### 2.3. Hand Gesture Recognition Based on FMG Sensors

FSRs have been used for hand gesture recognition often in recent years, sometimes alone [19], sometimes in combination with sEMG [20] or other sensors [21]. In these studies, the sensors were mostly worn on the forearm or the wrist [14]. In some rare cases, the sensors were worn while attached to a glove [20]. Moreover, FSR-based hand gesture recognition studies have practically focused on grasping [22], upper-arm activities such as pinching or rotations [21], and robotic hand [23] or prostheses control [24]. Moreover, there have been studies comparing grasp vs. nongrasp gestures [19]. However, sign language recognition have rarely been investigated with FMG signals and have never been the focus of any published scientific work except a few [18,25,26], where the feasibility of sign language recognition by FMG-based systems and investigations about the measurement system and the recognition with classic classification methods were conducted. Many studies have shown the advantages of FMG over EMG signals [19,27]. For example, FMG does not require much skin preparation and is less affected by skin impedance or sweat. Furthermore, FMG is characterized by its stability and robustness to external electrical noise; in addition, it does not necessitate the same amount of signal processing, and feature extraction as EMG [19]. Thus, all of these factors were the main reason for making the implementation of FMG in wearable devices more reliable in terms of cost and equipment. The oldest research discussing FMG features is from 2017 [28], while most research has implemented FMG as raw signals for gesture recognition. The discussed features for force myography are primarily used in grasping detection, robot hand control, and gait analysis [28,29,30,31,32]. Many researchers have achieved hand gesture recognition based on various machine learning methods. In addition, the hand gesture term includes a massive number of gestures with different levels of force and acceleration from sign language alphabets that generally cover postures and some slight motions to grasping and upper-arm activities that contain the interaction with objects and a high level of muscle contraction force. As for the different myography measurement techniques considered in this work, the high force level ensures a higher representation of the gesture. Most hand gesture recognition studies in the literature have focused on grasping and upper-arm activities. In contrast, sign language recognition is still an application where more investigations for features and classification methods are mandatory. Hence for the experimental part of this work, the application focus is on sign language recognition and, more specifically, American Sign Language (ASL) recognition. An overview of publications discussing American Sign Language recognition based on FMG as a standalone system or combining FMG and sEMG are listed in Table 2.

For FMG-based hand gesture recognition studies in the state of the art, the number of sensors is relatively high for portable and user-friendly systems. Moreover, the use of raw FMG signals in most of the studies limits the signal abilities and the machine learning methods’ performance. In addition, the applications of FMG are mainly focused on grasping and robotic hand or prosthesis control where a significant muscle contraction force is included, and they are rarely investigated for sign language recognition. From Table 2, for force myography, only one publication presented the sign language recognition by FMG as a standalone system based on commercial sensors [19]. However, that previous study was based on raw FMG only. In our studies in [18,25,35], the feasibility of sign language recognition by a reduced number of FMG sensors up to four and the investigation of various features for the recognition from an FMG-based bracelet with classic classification methods were conducted. In [25], it was proved that for a low-level ambiguity in the gesture set that the ELM could recognize the signs with an accuracy of 89.65% based on six standard features extracted from signals collected by four commercial sensors. In [35], the ELM accuracy for sign recognition based on the same extracted features as in [25] from the FMG signals collected by six sensors was equal to 91.11%. In this work, an optimized classifier is proposed, and its adequate minimal number of sensors to recognize various sets of signs with different levels of ambiguities is investigated.

## 3. Proposed k-Tournament Grasshopper Extreme Learner

The ELM has been proven in the literature to outperform other algorithms in terms of accuracy, speed, and model size. Therefore, it is more suitable for embedded systems. However, the weights’ random tuning remains a source of incertitude in terms of the optimal result this algorithm could reach. Researchers with different approaches proposed many optimizations of ELMs to reduce this effect. However, the used optimization methods were relatively old algorithms in the field. New optimization methods with good performances in various applications have been newly proposed and could give better results. Moreover, none of the proposed methods investigated the selection of randomly generated weights to optimize the architecture of an ELM without controlling its randomization process.

### 3.1. ELM Weights Selection

**Figure 1:**
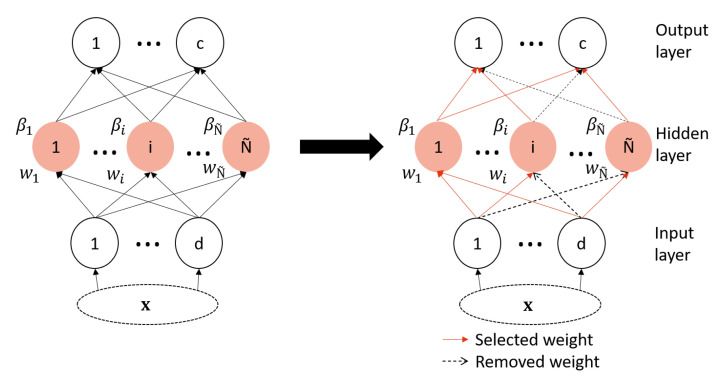
Proposed ELM architecture optimization strategy.

A neural network weight selection is one of the pruning types of network architecture, also named connection pruning, where the number of connections in the network is reduced. Another type is node pruning, where the number of hidden nodes is reduced by selecting the more significant hidden nodes [36]. For ELM pruning, researchers have proposed several methods for node pruning [8,37,38,39,40,41,42], but the weight pruning problem has not yet been studied. To cover this gap in the ELM architecture optimization strategies, a weight selection of the ELM is proposed in this paper as shown in Figure 1.

The selection of initially generated weights proposed in this work has the aim of keeping only the best subset of weights, which shares the same idea as other feature selection methods. In the latter methods, the goal, in general, is to define the best subset of features to improve the performance of the classification stage. Moreover, feature selection is important because the quality of the classification is variable along with the number of features. Hence, controlling this number is important because when it is too small, it may cause an overlap of data, which means it is not enough for the classification. However, if the number of features are too great, the dimension of the problem increases, and more complex classification algorithms are needed. Similar to the number of features, the number of weights in the ELM also impacts the overfitting, the model size, and the complexity. From there comes the inspiration to use a feature selection approach as a strategy for ELM weights’ selection.

### 3.2. k-Tournament Grasshopper Extreme Learning Machine for Selection Problems

First, the tournament process is included in the grasshopper repositioning process, as shown in the pseudocode in Algorithm 2 by controlling the best population evaluation.
**Algorithm 2:** k-Tournament grasshopper optimization algorithm.
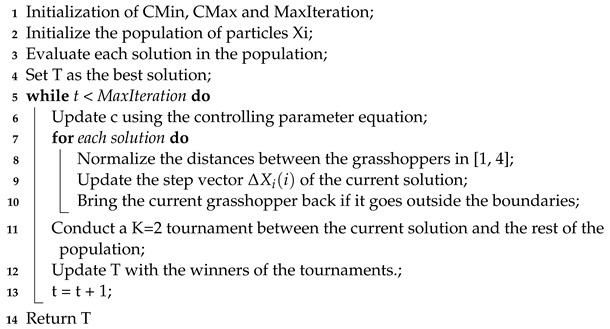


Furthermore, to perform the selection of this algorithm, the S-shaped transfer function is applied to the velocity of the search agents in the same way shown in the binary grasshopper optimization algorithm proposed in [43] presented by the pseudo-code in Algorithm 3 before combining it with the extreme learning machine shown in the Algorithm 1 as the wrapper’s evaluation classifier.

For this wrapper, the ELM was chosen as the evaluation method of the selected subsets because it outperformed other classification methods customarily used for wrapper building, such as KNN and SVM, in terms of accuracy, speed, and minimal computation complexity [44,45,46,47,48]. Moreover, searching for the best feature subset in feature selection is a challenging problem, especially in wrapper-based methods. This is because the selected subset needs to be evaluated by the learning algorithm (e.g., classifier) at each individual optimization step. Hence, a proper optimization method is required to reduce the number of evaluations, which is ensured by the ELM’s ability to solve multiclass problems in one iteration [49].
**Algorithm 3:** Binary grasshopper optimization algorithm (BGOA) [43].
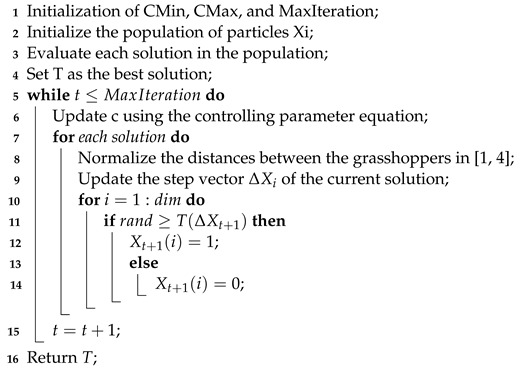


### 3.3. k-Tournament Grasshopper Extreme Learner

The final proposed KTGEL is shown in Algorithm 4. The proposed approach tends to optimize the extreme learning machine by selecting the most significant weights from the randomly generated ones during its initialization. The weight selection is integrated into the training process of the ELM. Moreover, the proposed KTGEL inherits the training procedure of the ELM, including the coupling between the input data and the input weights. Hence, the KTGEL is able to perform the feature selection within its training phase as an effect of the weight coupling relation with the input data during this phase. Each weight is coupled to one feature, but one feature is coupled to many weights, resulting in a feature being only eliminated if all its related weights are eliminated. Hence the proposed k-tournament grasshopper extreme learner is estimated to provide a better classification accuracy than the original ELM classifier on different biosignal databases for hand gesture recognition with a smaller model size as nonselected weights are replaced by zero so that no more computations are devoted to them.
**Algorithm 4:** Pseudocode of the proposed k-tournament grasshopper extreme learner.
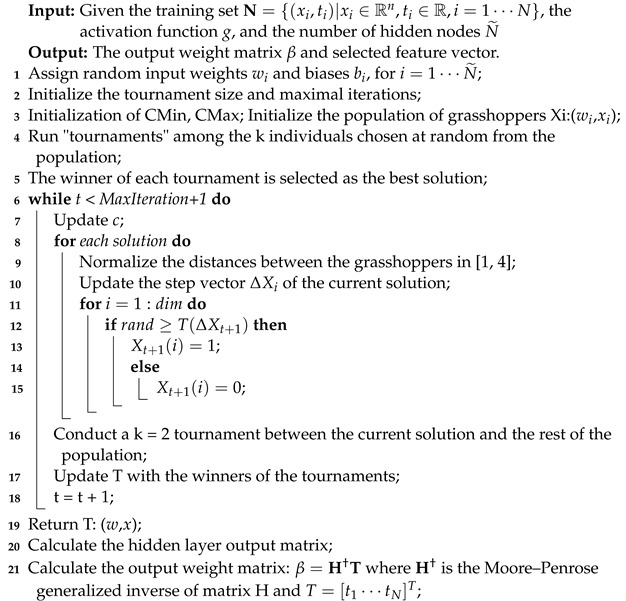



## 4. Experimental Investigations

In this section, three performance metrics were investigated: the accuracy in comparison with other works in the state of the art, reproducing the same set of gestures performed with the same number of subjects under as many similar conditions as possible, the influence of the number of sensors in relation with the number of subjects on the classification accuracy, and the influence of the ambiguity level in the set of gestures in comparison with an ELM after a feature selection step. The ELM and KTGEL were initialized with 3000 hidden nodes and compared on the data collected from eight FSR sensors with a total of 48 initial features in terms of accuracy, the final network architecture after weight selection by the KTGEL, the average sensitivity, and the average precision. To investigate the effect of the ambiguity level between gestures on their classification based on the FMG eight-sensor band, 40 participants from both genders in the age range between 20 and 32 years old participated in the collection of the 27 letters, the ASL numbers from 0 to 10, and the expression “I love you.”. Each subject participated in collecting only 10 or 9 signs with ten repetitions for each. In total, the collected data included 39 signs from the ASL, with 100 observations for each one. From this database, two sets of gestures were exploited in this paper for the investigations of the ambiguity level influence on the KTGEL performance. As for the evaluation with the accuracy, both the micro precision and the micro recall are conventionally used for a multiclassification assessment, where ${TP}_{j}$, ${FP}_{j}$, ${FN}_{j}$ are, respectively, the numbers of true positives, false positives, and false negatives of a class *j*, to show the overall classifier precision and sensitivity [50].

(2) $$\mathrm{micro}-P=\frac{\sum_{j=1}^{m}{\mathrm{TP}}_{j}}{\sum_{j=1}^{m}{\mathrm{TP}}_{j}+{\mathrm{FP}}_{j}}$$

(3) $$\mathrm{micro}-R=\frac{\sum_{j=1}^{m}{\mathrm{TP}}_{j}}{\sum_{j=1}^{m}{\mathrm{TP}}_{j}+{\mathrm{FN}}_{j}}$$

### 4.1. Comparison with the State of the Art of FMG-Based Gesture Recognition

In Table 3 a comparison between this work and the 2 studies from the state of the art was conducted to compare the algorithms’ performance while keeping the number of sensors, observations, and subjects. In [33], the FMG signals were collected with eight self-produced sEMG-FMG colocated sensors placed on the forearm of the subjects, and in this work, eight commercial FSR sensors were integrated into a wristband. In [34], carbon-nanotube-based FMG sensors were customized to produce more sensitive sensors with a higher ability to detect signs than commercial FSR sensors.

For FMG, the comparison with [33] showed that the proposed FMG bracelet located on the wrist and commercial sensors could provide better accuracy for ASL numbers’ classification. Moreover, the comparison with [34] was made with the exact same gestures proving that the KTGEL outperformed the ELM in terms of accuracy, even while implemented on data collected with commercial sensors. In contrast, the data in [34] were collected with optimized sensors that had been proved to outperform the commercial FSR sensors when the same signal processing was applied to data collected by both sensors.

### 4.2. Investigation of the Sensors and Subject Number Influence

**Figure 2:**
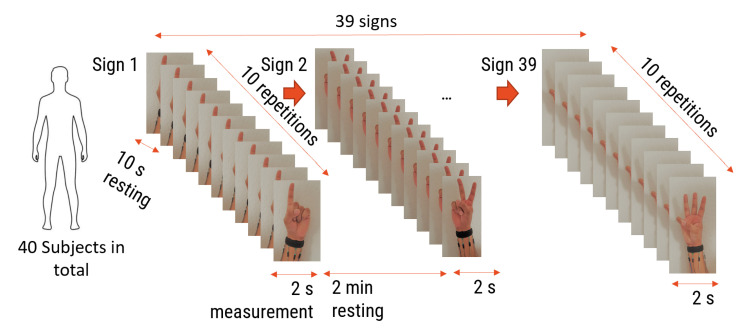
FMG signal collection protocol.

Force myography is rarely used for sign language recognition, and it has only been used with raw signals. Thus, it has not been sufficiently investigated in the literature. That is why it was necessary to conduct tests and observe the results. Both the number of sensors and the convenient features should be examined in this part. The idea is to find the optimal number of sensors from the wrist sensor band since previous studies [19] confirmed that the wrist-positioned band had more sensitivity to ASL than forearm bands. To minimize the sensors’ number and thereby ensure user comfort, two bands of six and eight commercial pressure sensors were designed, realized, and tested to find the band that led to the best accuracy for the ASL gesture recognition system. In the first band, eight sensors were placed with a gap of 2 cm around the wrist, while the second band had six sensors with a 2.25 cm gap between sensors. In all systems, Teensy boards with synchronized ADCs were employed as acquisition boards with a sampling frequency of 100 Hz. The two-band system was used to collect data during the performance of ASL signs according to the measurement protocol in Figure 2.

**Figure 3:**
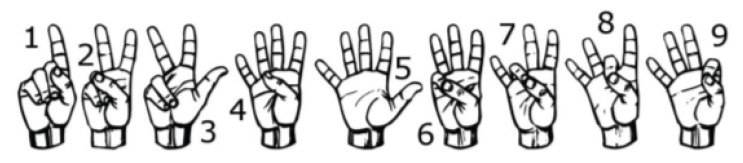
Performed ASL numbers from 1 to 9.

The first investigation aimed to test the feasibility of finger sign detection by the wrist FSR bands, including a small number of sensors compared to the state of the art, where the previous studies that implemented sign language included 16 commercial sensors [19] or 8 customized sensors [33,34]. Hence, only one person was asked to wear one of the two bands each time and perform the nine ALS numbers from one to nine shown in Figure 3 for twenty trials each.

Gestures have been performed with a resting of two minutes between every two gestures to avoid muscle fatigue. The collected signal seemed to have stationary behaviors for the different gestures, so it was estimated that even though features increased the performance of algorithms in comparison with raw data, there was no need for complicated features. Hence, six basic features, which were the min, max, RMS, var, STD, and mean, were extracted and normalized by the min–max method, and the KTGEL was used to classify the gestures. The classification accuracy was considered here as the evaluation criterion for the needed number of sensors for further data collection.

**Figure 4:**
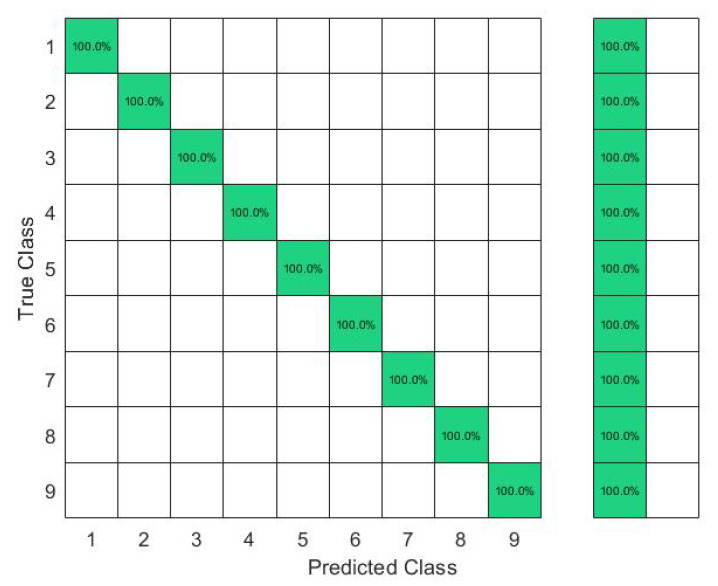
Confusion matrix of the KTGEL for one person for ASL numbers from 1 to 9:6 sensors.

**Figure 5:**
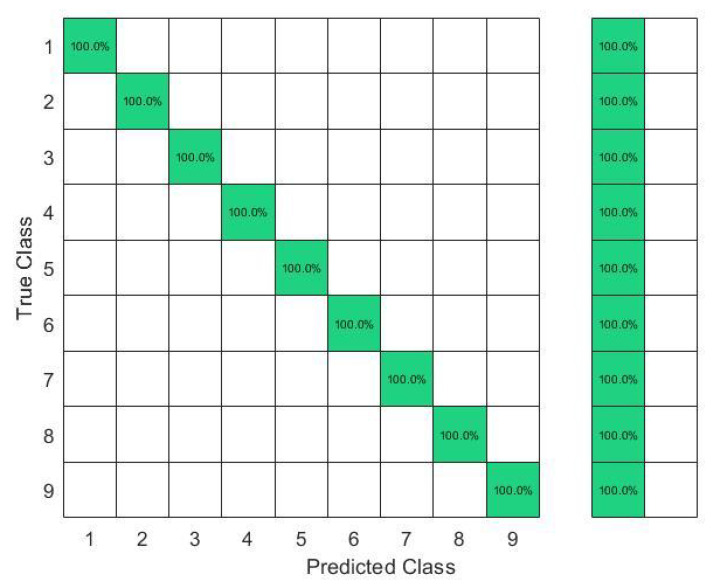
Confusion matrix of the KTGEL for one person for ASL numbers from 1 to 9:8 sensors.

In this set of gestures, the ambiguity level between signs could be described as low since no dynamic gestures were considered, and the similarity between the gesture performance process was limited between the numbers six, seven, eight, and nine between all the possible combinations of the nine gestures. The collected data from only one person resulted in a total of 180 observations. In this investigation, 80% of the observations were used to train and validate the KTGEL using a fivefold cross-validation while saving a random 20% of each gesture’s data to be used only as testing data. From the confusion matrices in Figure 4 and Figure 5, it could be confirmed that for only one subject performing the gestures, both bracelets could detect and allow the classifier to predict the nine tested gestures correctly. It was proved by this investigation that the six sensors were sufficient to recognize gestures with a low ambiguity level collected from only one subject.

The second investigation was to evaluate the system’s stability and accuracy for the same gesture recognition while increasing the number of subjects to 10 subjects. However, in that investigation, each person was asked to perform each gesture only ten times. In total, 1000 observations were used in this implementation of the KTGEL, with 80% of the observations employed to train and validate the model using fivefold cross-validation while a random 20% of each gesture observations were safeguarded to be used only as testing data.

**Figure 6:**
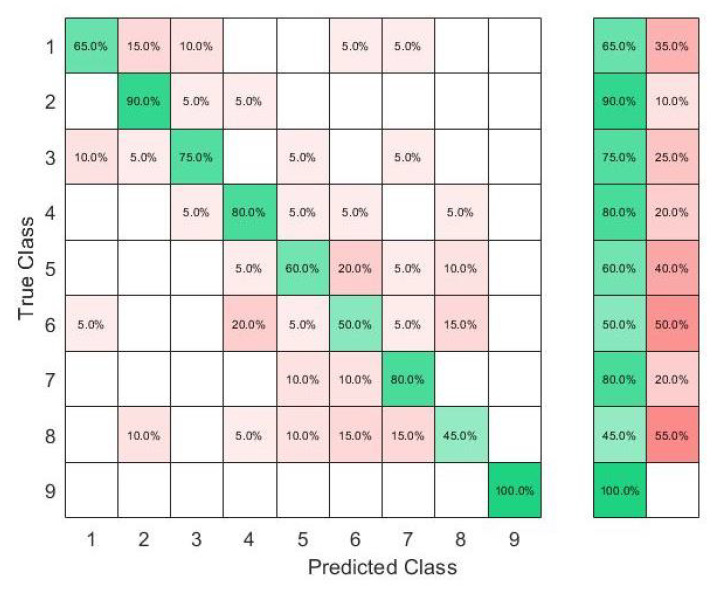
Confusion matrix of the KTGEL for ten person and nine numbers: 6 sensors.

**Figure 7:**
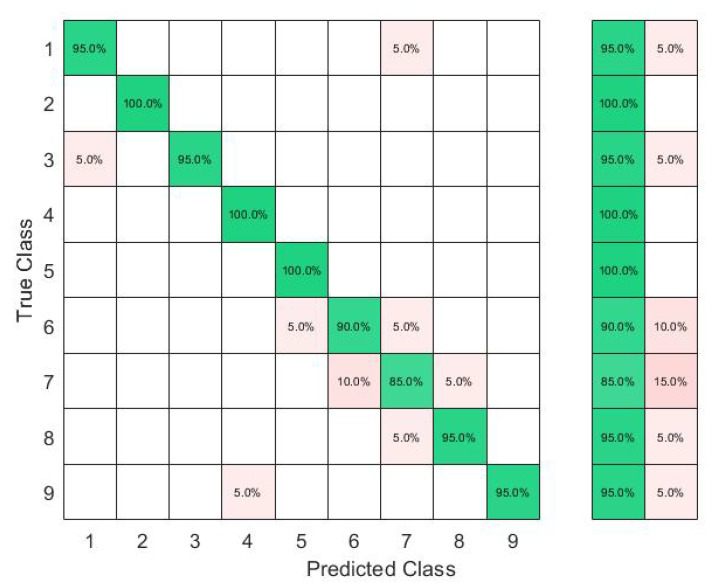
Confusion matrix of the KTGEL for ten person and nine numbers: 8 sensors.

The results in Figure 6 show that with six sensors collecting nine gestures, the KTGEL had a test accuracy of 71%. Figure 7 shows that the eight sensors band collecting American Sign Language numbers could be recognized with an accuracy of 95%. These results confirmed that six sensors were not suitable enough for FMG-based gesture recognition with several subjects. The additional complexity in the signals induced by the physiological difference between the various subjects could not be canceled by the use of six sensors only. It is also observed in Figure 6 that the confusion between gestures could not be totally obvious from the gestures’ nature, which led to the estimation that the collected data were not enough to differentiate the gestures. However, observing Figure 7, it could be seen that the confusions were limited, with the most relevant confusions being between gestures six, seven, and eight. Hence, this investigation showed that eight FSR sensors as the minimal number of sensors had an acceptable gesture recognition accuracy from the data collected from 10 subjects. In addition, to confirm the user’s comfort with the used number of sensors, subjects were asked about their evaluation of the band. None of the subjects complained about the sensor band placement, but they announced that the material used for the actual band was not soft enough. Hence, the eight-sensor band was kept for further data collection as a possible standalone system for a future investigation of sign recognition with more features, and a modification of the bracelet material will be considered as an outlook of the system design.

### 4.3. Recognition of ASL Signs with a Middle Ambiguity Level

**Figure 8:**
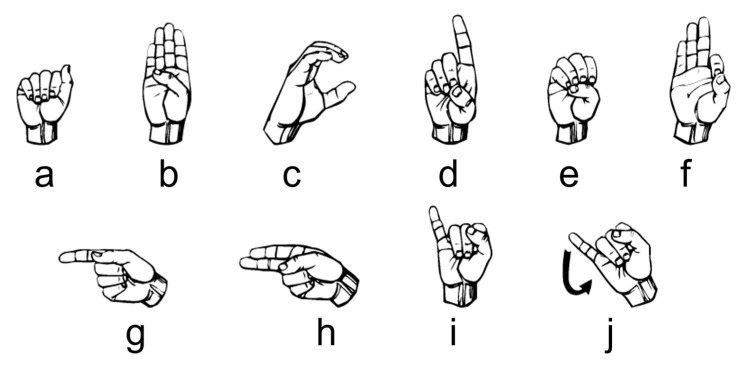
Ten ASL letters, A–J.

To investigate the influence of ambiguity on the KTGEL performance for American Sign Language recognition, the first ten alphabet letters from A to J were collected from 10 healthy subjects. During the data collection, subjects followed an informative video for ASL teaching. Gestures were collected as postures except for the letter J, which was a dynamic gesture including a rotation movement of the wrist as symbolized by the arrow in Figure 8. This set of gestures was considered to have a middle ambiguity level as it included a dynamic gesture and a similarity in the posture between the signs A, C, and E and the signs G and H.

Implemented with 3000 hidden nodes after a feature selection by the KTGELM, the ELM had both a microaverage precision and a microaverage sensitivity of 97% when trained with only 13 selected features out of the original 48 features, as it is detailed in the comparison presented in Table 4. The KTGEL initialized with 3000 hidden nodes resulted in a trained model with only 1000 hidden nodes while it was given the full 48 features as inputs. The KTGEL reached the same total sensitivity with a reduced total precision by only 1% in comparison with the ELM after a separate feature selection stage.

**Figure 9:**
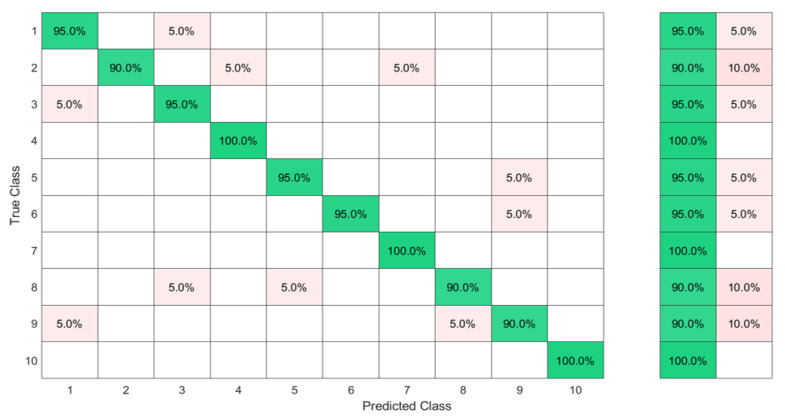
Ten ASL letters, A–J, detected with FMG and classified by the ELM after feature selection by the KTGELM.

**Figure 10:**
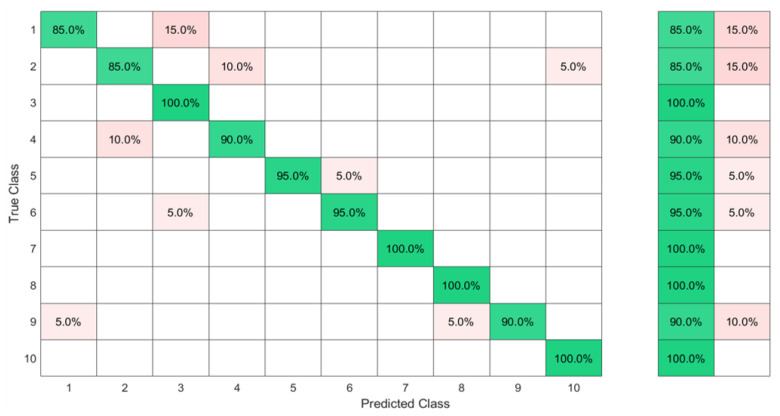
Ten ASL letters, A–J, detected with FMG and classified by the KTGEL.

The confusion matrices in Figure 9 and Figure 10 show that even though J was a dynamic gesture, it was 100% recognized using the FSR wrist band, which could be explained by the muscle deformation resulting from the rotation of the wrist which resulted in a stronger level of the signal in comparison with the other signs where the muscle movements in the wrist level were not visible.

### 4.4. Recognition of ASL Signs with a High Ambiguity Level

**Figure 11:**
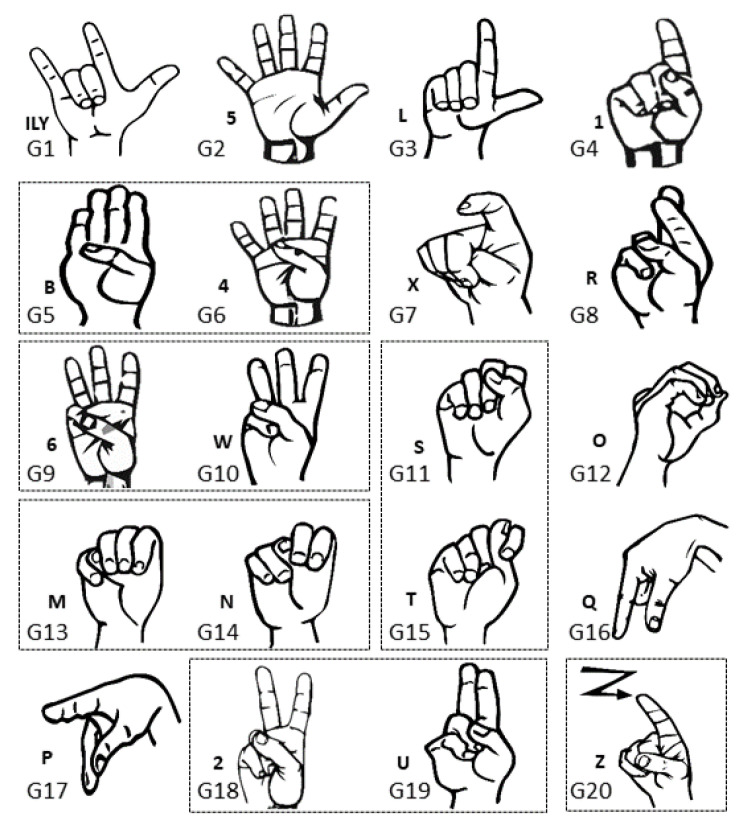
Data set of 20 ASL letters with expected high ambiguity, namely between “B” and “4”, “M” and “N”, “U” and “2”, “6” and “W”, “S” and “T”, and a dynamic sign “Z”.

The used data set in this part included the 20 ASL letters shown in Figure 11 with 10 of the signs showing a big similarity, namely between “B” and “4”, “M” and “N”, “U” and “2”, “6” and “W,” “S” and “T”, and a dynamic sign “Z”, so the expected accuracy could be as low as 50% for this data set.

**Figure 12:**
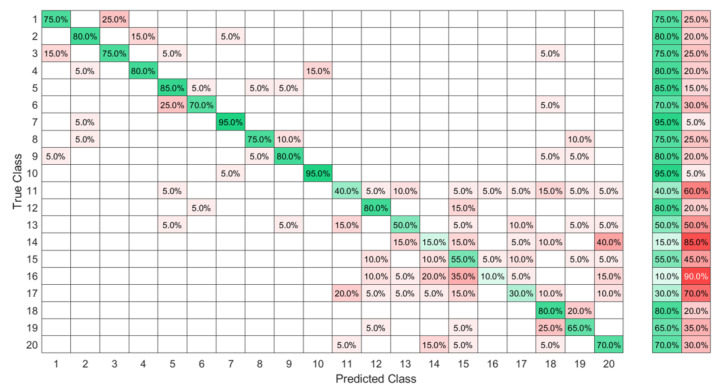
High-ambiguity data set classified by ELM after feature selection by the KTGELM.

The same sign set was collected by 20 new subjects while wearing the eight-sensor band, and 100 observations of each sign were collected as FMG data. The data collected by the FMG sensor at the wrist level presented not only information about muscle contraction but also about the tendon state. As the sensors were distributed around the wrist, the FMG band could cover all the superficial muscles. Hence, more confusion between signs was noted due to the force transmission through the muscle fibers during the contraction and the influence of the deep muscle on the superficial ones. Therefore, different signs could have the same FMG response at the level of one or more sensors when signs shared an initial hand shape or the same performing fingers. For the ELM after the KTGELM feature selection shown in Figure 12, it could be seen that the signs “T” and “S”, symbolized as classes 15 and 11, presented a source of confusion for the rest of the signs as not only the majority of their observations were misclassified, but also many other classes were mispredicted as signs “T” and “S”. Based on the FMG data set, the ELM after feature selection by the KTGELM presented a classification microaveraged precision of 78% with a sensitivity of 80% among the 20 signs.

**Figure 13:**
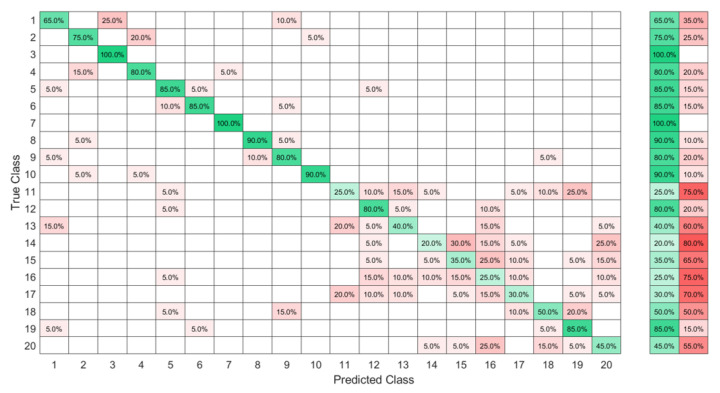
High-ambiguity data set classified by the KTGEL without a previous feature selection.

Using the same database, the KTGEL resulted a trained model with 1000 hidden nodes. The original data without feature selection are presented in the confusion matrix in Figure 13, where similar results to the ELM with feature selection could be seen with a micro-p of 77% and a micro-r of 80% and a thrice smaller model size. Evaluating the overall classification accuracy, it could be seen that the ELM after the KTGELM and the KTGEL had the same performance in most cases, with the second being less complicated as it had only 1000 hidden nodes and could do the feature selection and the classification in the same process.

## 5. Conclusions

This work focused on recognizing American Sign Language based on commercial FMG sensors. We proposed to optimize an ELM by a weight-pruning method to optimize the network architecture and maintain the randomness of the initial weights. The pruning reduced the network size in the ELM by removing the weights, which were participating less in the classification result. We proposed to use the k-tournament grasshopper optimization algorithm (KTGOA) as the core of the ELM’s weight-pruning process due to its fast convergence in multidimensional optimization spaces. A KTGOA was implemented to select the ELM weights. Thereby, a k-tournament grasshopper extreme learner (KTGEL) was proposed as a classifier with a reduced architecture, high performance, and internal feature selection. The influence of the number of FMG sensors and the number of subjects on the performance of the KTGEL was first investigated. It was proved in this paper that if only one subject was performing the data collection, a six-sensor bracelet was sufficient. However, with an increasing number of subjects, eight sensors were the minimal number needed to recognize the ASL numbers accurately. The investigation of the influence of the ambiguity level in the set of gestures on the performance of the KTGEL compared with the ELM showed that both had similar accuracy in the case of middle and high ambiguity levels. However, the ELM was trained with fewer features as it was preceded by a feature selection wrapper, while the KTGEL was trained with all the features. Moreover, in both tested cases, the KTGEL-trained model reduced the number of initially hidden nodes by two-thirds. The KTGEL also showed similar sensitivity and precision values with those of the ELM trained with selected features. The proposed KTGEL was created by the KTGOA that optimized the process of the best solution selection but inherited the linear behavior of the exploration–exploitation balancing coefficient from the original GOA. Similarly to the GOA, this linearity could lead to a trapping into a local optimum during the selection process of coupled features and weights, during the weight pruning in the KTGEL. Hence, in future work, the nonlinearization of the exploration–exploitation coefficient for the weight selection process will be investigated.

## Figures and Tables

**Table 1:** FSR Interlink and Flexiforce properties from data sheets [14].

Title 1	Interlink FSR (FSR402)	Flexiforce (FLX-A201-F)
Minimum actuation force (N)	0.1	N/A
Force sensitivity range (N)	0.1–10	0 to 4.4, 0 to 445
Single-part force repeatability	±2%	±2.5%
Part-to-part force repeatability	±6%	±40%
Drift	<5% per log10 (time)	<5% per log10 (time)
Hysteresis	+10%	<4.5%
Response time (μs)	<3	<5
Linearity error	N/A	<±3%

**Table 2:** State of the art for FMG-sensor-based ASL recognition.

Sensors	Features	Subjects	Gestures	Classifier	Accuracy
8 colocated	MAV, WL	5	10	LDA	91.6%
sEMG FMG	ZC, SSC				
[33]					
8 FMG	MAV	5	10	LDA	80%
Self-produced					
[33]					
16 FMG	RAW signal	12	16	LDA	96.70%
[19]					
8 nanocomposite sensors	min, max, mean, RMS, median, STD	10	10	ELM	93%
[34]					

**Table 3:** Performance of the proposed classifiers vs. the state of the art of ASL numbers’ recognition by FMG.

Work	Hand Signs	Sensor No.	Sensor	Classifier	Accuracy in %	Observations	Subjects
[33]	10	8	Customized	LDA	80.00	50	5
This work	10	8	FSR	KTGEL	88.00	50	5
[34]	10	8	Customized	ELM	93.00	100	10
This work	10	8	FSR	KTGEL	98	100	10

**Table 4:** Comparison between the ELM and KTGEL in recognition of ASL signs with a middle ambiguity level.

	ELM	KTGEL
Additional feature selection algorithm	yes	no
Initial number of features	13	48
Initial number of hidden nodes	3000	3000
Final number of hidden nodes	3000	1000
Training time with feature selection in seconds	9.5	2.5
Testing time in seconds	0.22	0.04
Testing accuracy in %	95	94
Precision in %	97	96
Sensitivity in %	97	97
